# The role of *Bifidobacterium* genus in modulating the neonate microbiota: implications for antibiotic resistance acquisition in early life

**DOI:** 10.1080/19490976.2024.2357176

**Published:** 2024-05-26

**Authors:** Anna Samarra, Raúl Cabrera-Rubio, Cecilia Martínez-Costa, Maria Carmen Collado

**Affiliations:** a Department of Biotechnology, Institute of Agrochemistry and Food Technology- National Research Council (IATA-CSIC), Paterna, Valencia, Spain; b Department of Pediatrics, School of Medicine, University of Valencia, Valencia, Spain; c Pediatric Gastroenterology and Nutrition Section, Hospital Clínico Universitario Valencia, Valencia, Spain

**Keywords:** *Bifidobacterium*, infant, antibiotic, resistance, microbiota, gut

## Abstract

Resistance to antibiotics in newborns is a huge concern as their immune system is still developing, and infections and resistance acquisition in early life have short- and long-term consequences for their health. *Bifidobacterium* species are important commensals capable of dominating the infant gut microbiome and are known to be less prone to possess antimicrobial resistance genes than other taxa that may colonize infants. We aimed to study the association between *Bifidobacterium*-dominated infant gut microbiota and the antibiotic resistant gene load in neonates, and to ascertain the perinatal factors that may contribute to the antibiotic resistance acquisition. Two hundred infant fecal samples at 7 days and 1 month of age from the MAMI birth cohort were included in the study and for whom maternal-neonatal clinical records were available. Microbiota profiling was carried out by 16S rRNA amplicon sequencing, and targeted antibiotic resistance genes (ARGs) including *tetM, tetW, tetO, blaTEM, blaSHV* and *ermB* were quantified by qPCR. Infant microbiota clustered into two distinct groups according to their *Bifidobacterium* genus abundance: high and low. The main separation of groups or clusters at each time point was performed with an unsupervised non-linear algorithm of k-means partitioning to cluster data by time points based on *Bifidobacterium* genus relative abundance. Microbiota composition differed significantly between both groups, and specific bifidobacterial species were enriched in each cluster. Lower abundance of *Bifidobacterium* in the infant gut was associated with a higher load of antibiotic resistance genes. Our results highlight the relevance of *Bifidobacterium* genus in the early acquisition and establishment of antibiotic resistance in the gut. Further studies are needed to develop strategies to promote a healthy early colonization and fight against the spread of antibiotic resistances.

## Introduction

Antibiotic resistance in infants is a growing concern. Early exposure to antibiotics can lead to the development of antibiotic-resistant bacteria and disrupt the delicate balance of the gut microbiome, having consequences for future infant health, immune development and metabolism. In the early stages of life, antibiotic acquired resistance can result from the horizontal transfer of genes between bacteria, vertical transmission from the mother at birth or during lactation, and/or antibiotic exposure of the infant.^1^

The gut microbiota composition is the main source of most antibiotic resistant genes (ARGs) that are carried by a limited number of taxa. Members of the family Enterobacteriaceae, such as *Escherichia, Klebsiella, Citrobacter*, and *Enterobacter*, responsible for many perinatal infections, contribute the most abundant and unique ARGs in the infant gut.^2^ *Bifidobacterium* are negatively correlated with antibiotic resistance load;^3^ these species are important commensals capable of dominating the gut microbiome of breastfed infants, in part because of their capacity to use human milk oligosaccharides (HMOs) and other carbohydrates efficiently.^4,5^ *Bifidobacterium* species are also less likely to possess ARGs than other taxa such as *Enterococcus, Streptococcus, Staphylococcus*, and *Bacteroides* genus.^6,7^ It has also been reported that the resistance genes detected in anaerobes (such us *Bifidobacterium* or *Lactobacillus*) are distinct from the ARGs found in pathogenic bacteria that grow aerobically,^8,9^ which suggest that horizontal gene transfer between commensals and pathogens is more unlikely than the transfer of ARGs between pathogens.^10,11^

The infant gut microbiota changes significantly during the first days and weeks of life, with external factors, such as breastfeeding, mode of delivery, antibiotic exposure, and diet, exerting notable influences on the composition and diversity of the infant gut microbiota, which in turn affects the resistome.^12^ Understanding these influences is crucial for developing interventions and strategies aimed at promoting a healthy gut microbiota and mitigating the risks associated with the emergence and spread of antibiotic resistance in infants. Our objective was to study the influence of the presence of *Bifidobacterium* spp. on the early gut microbiota and the acquisition of antibiotic resistance gene load in neonates from 7 days to 1-month old. Further, we examined which perinatal factors may contribute to the initial microbiota composition and antibiotic resistance acquisition.

## Materials and methods

### Study design and volunteers

A total of 200 infant fecal samples collected during the first month of life of healthy term (37 weeks or more) infants from the MAMI birth cohort,^13^ recruited during 2015–2017, were included in this observational study (Supplementary figure S1). Maternal-infant clinical records including body mass index (BMI), gestational age, neonate gender, birth weight, mode of delivery, type of lactation (maternal lactation, referring to breastfeeding; mixed lactation, referring to combination of both breastfeeding and formula-feeding; and exclusive formula-feeding) and antibiotic exposure of the infant were collected. All participants received oral and written information about the study, and written consent was obtained. The study was approved by Ethical Committee of Hospital Clínico Universitario de Valencia and CSIC. The trial was registered on the ClinicalTrial.gov platform (number NCT03552939).

### Neonatal biological samples and DNA extraction and quantification

Samples were collected at two time points (7 days and 1 month after birth) in sterile containers by the parents at home following detailed instructions. They were immediately stored at −20°C, transported within 24 h to primary health centers and stored at −80°C until analysis. Sample management and processing, as well as Total DNA was extraction, purification, and concentration were performed as described previously.^14^

### Quantification of antibiotic resistance genes and Bifidobacterium spp. By targeted qPCR

To analyze the antibiotic resistance gene load in infants’ gut, a subset of samples from each time point (7 days: *n* = 71, 1 month: *n* = 66) was used, and the presence and abundances (log copies/µl) of each ARG were compared between the two time points and the two *Bifidobacterium* relative abundance clusters. Quantifications of the total number of copies of genes conferring resistance to tetracycline (*tetM, tetO* and *tetW)*, beta-lactam (*blaTEM* and *blaSHV*) and erythromycin (*ermB*) were performed using described primers (Supplementary Table S1) by qPCR in a LightCycler 480 real-time PCR system (Roche Technologies, Basel, Switzerland). The quantified ARGs were chosen according to their importance and high prevalence in infant resistome as described elsewhere.^2,3,15–18^ Each reaction mixture of 10 μL was composed of 5 μL SYBR GreenPCR Master Mix (Roche Technologies, Basel, Switzerland), 0.25 μL of each of the primers at a concentration of 10 μM, 3.5 μL of distilled water and 1 μL of DNA. The amplification conditions were 35 cycles of: initial denaturation at 95°C for 5 min denaturation at 95°C for 30 s, specific annealing temperature for each primer pair^19^ for 30 s, and a final extension step of 72°C for 30 s. The qPCR results were calculated as the antibiotic resistance gene copies per sample (log copies/µl). *Bifidobacterium* genus total counts were also determined by targeted quantitative PCR by the same quantitative system based on the amplification of specific 16S rRNA gene region. Melting curves were also assessed to test the specificity of the reaction. For all qPCR measurements, standard curves for the specific targeted gene were generated using Ct values and the calculated gene copy numbers were determined based on the fragment amplification length.

### Gut microbiota profiling by specific 16S rRNA amplicon sequencing

The microbiota characterization was performed by amplification of the V3-V4 regions of the 16S rRNA. Amplicons were obtained with PCR amplification using barcoded conventional primers (341F 5′-CCTACGGGNGGCWGCAG-3′ and 806 R 5′GGACTACNNGGGTATCTAAT-3′) with a 466bp fragment length. Amplicons were checked with a Bioanalyzer DNA 1000 chip and libraries were sequenced using a 2 × 250 bp paired-end kit on a MiSeq-Illumina platform (FISABIO sequencing service, Valencia, Spain). Bacterial diversity analysis was done using raw reads, which were quality controlled and filtered (Quality; 25 and length; 125 bp) using TrimGalore (v0.6.4_dev; https://github.com/FelixKrueger/TrimGalore).

The paired-end reads with a minimum overlap of 30 bp were joined using Fastq-join.^20^ Sequences were trimmed of primers and distal bases, and singletons were removed with USEARCH v11.^21^ zOTUs (zero-radius operational taxon units) mapping to the human genome (GRCh38) using the Burrow – Wheeler Aligner in Deconseq v0.4.3 were filtered out. The resulting reads were denoised, and chimeras were filtered with UNOISE3.^22^ Taxonomic assignment of zOTUs was performed in QIIME 2 v2018.2^23^ using the QIIME 2 feature classifier plugin^24^ and the Ribosomal Database Project (RDP).^25^ The zOTUs were aligned with MAFFT^26^ to then make a phylogenetic tree with FASTTREE^27^ that was midpoint rooted. The Bayesian LCA-based Taxonomic Classification Method (BLCA)^28^ was used to assign zOTUs to species that were only assigned to *Bifidobacterium* genus with QIIME 2 (assignation of 80% confidence). The database used was a custom database, taking the reference sequences of all *Bifidobacterium* species from the SILVA database.^29^ We filtered the zOTU data using filter_taxa function of “*phyloseq*” package, and only zOTUs present in at least 10% of samples were retained.

### Bioinformatic and statistical analyses

All statistical analyses were performed with R version 3.6.0, and figures were drawn with the “*ggplot2*” R package.^30^ Categorical variables are expressed as positive cases-prevalence and (percentage, %). Normally distributed data are presented as mean ± standard deviation (SD) and non-normal data as median and interquartile range [IQR]. Pearson’s-Chi-square test was used for categorical variables, and Mann–Whitnney U test or Fisher’s exact test were used for continuous variables, as appropriate, for calculating statistical significance. Statistical analyses were performed equally for both paired and unpaired data points, as the majority of the population was paired in both time points, and confirmation analyses confirmed there were no differences (data not shown).

The main separation of groups or clusters at each time point was performed with an unsupervised non-linear algorithm of k-means partitioning to cluster data by time points based on *Bifidobacterium* genus relative abundance. The elbow method and average silhouette method were performed using in the “*factoextra*” R package^31^ were used to determine the optimal number of clusters and k-means clustering of the data was performed with the “*stats*” R core package. Optimal centers of k-means were established as 2 clusters, which corresponded with high and low *Bifidobacterium* relative abundance (Supplementary figure S2).

Venn diagrams were generated to assess the core and unique microbiota, at the zOTU level, for the two clusters at each time point separately, using the R package “*VennDiagram*”.^32^ Calculations of richness (Observed, Chao1 and ACE) and evenness indices (Shannon and Simpson) were done using the “*phyloseq*” R package.^33^

Beta diversity was characterized by Principal Coordinate Analysis (PCoA) conducted by plotting the Bray-Curtis distance matrix of log transformed zOTU counts for each time point separately. The Adonis permutational test was used to evaluate overall differences in microbiota structure between the k-mean-categorized groups with the “*vegan*” R package.^34^ The same analysis was performed according to the mode of delivery and antibiotic exposure of the study participants. A Mann – Whitney U test in the “*rstatix*” R package^35^ was performed on the log transformed abundances of genera to identify significantly different genera between the two clusters. Phylogenetic trees based on zOTUs assigned to *Bifidobacterium* genus were constructed using “*plot_tree*” function of “*phyloseq*” package.

Multivariate Redundancy Analysis (RDA) was conducted to confirm the correlation between ARGs and microbiota with respect to the clusters of *Bifidobacterim* abundance. Linear discriminant analysis effect size (LEfSe) was used to identify microbial genera significatively enriched in each cluster. An LDA score (log10) > 3 was considered significant. Spearman rank correlation coefficients were estimated to determine the linear association between the 20 top genera and the number of copies of ARGs.

The effect size and significance of each covariate were determined using the ‘*envfit’* function in the “*vegan*” package comparing the difference in the centroids of each group relative to the total variation. Ordination was performed using NMDS based on Bray – Curtis dissimilarity. The significance value was determined based on 999 permutations. The squared correlation coefficient (R^2^) for each variable was calculated and scaled as a vector. In total, 6 covariates with known associations to gut microbiome development in neonates, infants, and children were included in the ‘*envfit*’ analysis and the grouping used within each variable is presented in Table 1. Specifically, we tested hierarchical clustering based on k-means according to *Bifidobacterium* relative abundance (high or low), mode of delivery, mode of lactation, antibiotic exposure during pregnancy, antibiotic exposure at birth, and direct antibiotic exposure of the infants. MaAsLin2^36^ was used to build general linear models for longitudinal data to efficiently determine the multivariate association between factors and taxonomy in both clusters while accounting for potentially confounding covariates. MaAsLin2 relies on general linear models to accommodate most modern epidemiological study designs, including cross-sectional and longitudinal. In this case, the analysis with MaAsLin2 was a longitudinal analysis, so the first added variable was Time (7 days and 1 month). The following variables were added depending on the strength of each variable seen in the other analyses, so we added “kmeans_ab02” (Low vs Hight), “Delivery_mode” (C-section vs vaginal) and “Lactation” (maternal vs. mixed vs. formula). No more variable groups or other variables were added. The command runs MaAsLin2 on the data, running a multivariable regression model to test for the association between microbial species abundance versus Time, kmeans_ab02, Delivery_mode and Lactation. Finally, the input data were transformed to log format.

**Table 1.** Characteristics of study participants.

	7-days-old infants (*N* = 99)			1-month-old infants (*N* = 101)		
	High (*N* = 42)	Low (*N* = 57)	*p*-value	High (*N* = 43)	Low (*N* = 58)	*p*-value
**Gender**						
Female	17 (40.47%)	32 (56.14%)	.181	21 (48.83%)	31 (53.44%)	.797
Male	25 (59.52%)	25 (43.38%)		22 (51.16%)	27 (46.55%)	
**Delivery mode**						
c-section	4 (9.52%)	21 (36.84%)	.004*	8 (18.6%)	17 (29.31%)	.317
Vaginal	38 (90.47%)	36 (63.15%)		35 (81.39%)	41 (70.68%)	
**Clinical data**						
Weight (kg)	3.38 ± 0.68	3.30 ± 0.45	.147	4.28 ± 0.49	3.99 ± 0.75	.527
**Lactation mode**						
Maternal	39 (92.85%)	50 (87.71%)	.843	30 (69.76%)	46 (79.31%)	.536
Mixed	0	1 (1.75%)		6 (13.95%)	6 (10.34%)	
Formula	3 (7.14%)	6 (10.52%)		7 (16.27%)	6 (10.34%)	
**Antibiotic exposure**						
At birth	4 (9.52%)	24 (42.10%)	.0008*	10 (23.25%)	18 (31.04%)	.523
During first 7 days	3 (7.14%)	9 (15.7%)	.321	6 (13.95%)	5 (8.62%)	.598
During 1st month	1 (2.38%)	5 (8.77%)	.372	3 (6.97%)	5 (8.62%)	1
**Pregnancy data**						
Gestational age (weeks)	38.88 (39–41)	39.38 (39–40)	.070	38.55 (38.25–40)	39.74 (39–40)	.180
Weight gain over the pregnancy (kg)	11.88 ± 4.09	12.47 ± 4.52	.397	13.02 ± 3.99	12.56 ± 5.21	.018
Pre-gestational BMI (kg m − 2)	22.33 ± 4.99	23.99 ± 3.57	.089	23.34 (4.75)	22.38 (3.28)	.07
Antibiotic consumption during pregnancy	7 (16.6%)	10 (17.5%)	.070	11 (25.58%)	19 (32.75%)	.575

Categorical variables are expressed as positive cases-prevalence and percentatge (%). Normally distributed data are presented as mean ± standard deviation (SD) and non-normal data as median and interquartile range [IQR]. Pearson’s-Chi-square test was used for categorical variables, and Mann–Whitnney U test or Fisher’s exact test were used for continuous variables, as appropriate, for calculating statistical significance. Asterisks (*) indicate a significant difference (*p* < .05) between two groups. BMI: Body Mass Index.

For all methods, p-values were adjusted for multiple comparisons using False Discovery Rate (FDR) based on Benjamini – Hochberg (BH).^37^ Normality of the data was evaluated with Shapiro – Wilk tests.

## Results

### *Bifidobacterium* dominance associates with a specific microbial composition

**Figure 1.**
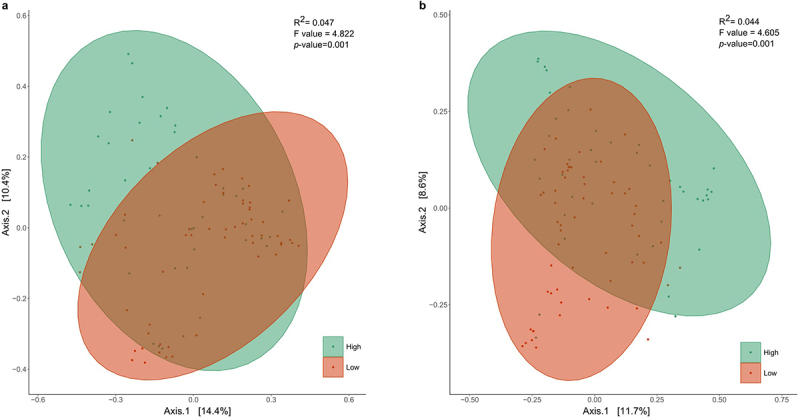
Principal coordinates analysis of the hierarchical clustering of *Bifidobacterium* relative abundance of the infants at (a) 7 days and (b) 1 month of age based on Bray Curtis distances regarding the microbial community composition (*p* < .001; ADONIS pairwise test). Samples are grouped by hierarchical clustering of *Bifidobacterium* relative abundance in intestinal microbiota. Orange ellipses represent the high-*Bifidobacterium* groups and blue ellipses represent low-*Bifidobacterium* groups. Supplementary figure 2 provides the optimal number of clusters found by the elbow and average silhouette methods of K-means partitioning clustering algorithm.

Hierarchical clustering revealed two significantly distinct (*p <* .001) microbial clusters at each time point, characterized by a high (54.38% for 7 days; 61.72% for 1 month) and low (11.26% for 7 days; 17.08% for 1 month) relative abundance of *Bifidobacterium* members (Figure 1). 71.43% of infants maintained this classification according to *Bifidobacterium* relative abundance through time (from 7 days to 1 months).

Clinical characteristics of the participating infants and their respective mothers at both time points according to *Bifidobacterium* cluster are shown in Table 1. Gender was distributed similarly between the two clusters. Regarding the mode of delivery, vaginal-born infants corresponded to 90.47% and 81.39% of the neonates clustered as high-*Bifidobacterium* at 7 days and 1 month, respectively. Most of the infants participating in the study were breastfed and no differences with other breastfeeding practices were found between clusters. Finally, antibiotic exposure was recorded during the gestation period until 1 month of age. 42.10% of infants clustered as low-*Bifidobacterium* at 7 days of age were exposed to antibiotics at delivery, significantly more than the high-*Bifidobacterium* infants (*p =* .0008). According to maternal characteristics, no statistical differences were found according to the *Bifidobacterium* cluster.

To assess if confounding variables might influence clustering, beta diversity was also compared across other metadata variables, separating the infants by their mode of delivery in combination with the two different clusters, corresponding to the *Bifidobacterium* relative abundance (Supplementary table S2). For both time points, vaginal-born infants clustered in two groups according to the *Bifidobacterium relative abundance* (Vaginal_High and Vaginal_Low) with significantly different bacterial communities (*p =* .006). The microbiota of c-section_Low infants was significantly different from Vaginal_Low and Vaginal_High infants (*p <* .05), both at 7 days and 1 month of age. Finally, in 1-month-old infants, C-section_High infants clustered separately from Vaginal_Low infants (*p =* .036). No differences between c-section-High and c-section Low were found at either time point.

The impact of antibiotic exposure microbial composition in the context of *Bifidobacterium* clustering was also examined (Supplementary table S3). Both high and low- *Bifidobacterium* groups of infants that did not receive antibiotics had different gut microbial compositions (*p =* .012 and *p =* .006 for 7 days and 1 month of age). Infants with a low abundance of *Bifidobacterium* have a significantly different microbial composition at 7 days of age depending on if they have received antibiotics or not (*p =*.012), which is not observed in high-*Bifidobacterium* cluster.

### Microbiota is shaped by Bifidobacterium spp. abundance

In both time points, the percentage of the core zOTUs was 35.1%, while 48.8% (7 days) and 50.5% (1 month) are unique for the Low cluster and 16.1% (7 days) and 14.1% (1 month) were unique for the High cluster, signifying that the two groups, harbor different inhabitant niches, independent of time.

Infants in the High cluster showed lower but not statistically significant microbial diversity and richness at 7 days and 1 month of age (Supplementary table S4). The observed diversity of *Bifidobacterium* species between the two clusters at the two sampling times was significantly different (*p* < .001 for both times): the high-*Bifidobacterium* group had more diversity of *Bifidobacterium* spp. than the low-*Bifidobacterium* group (Supplementary figure S3).

**Figure 2.**
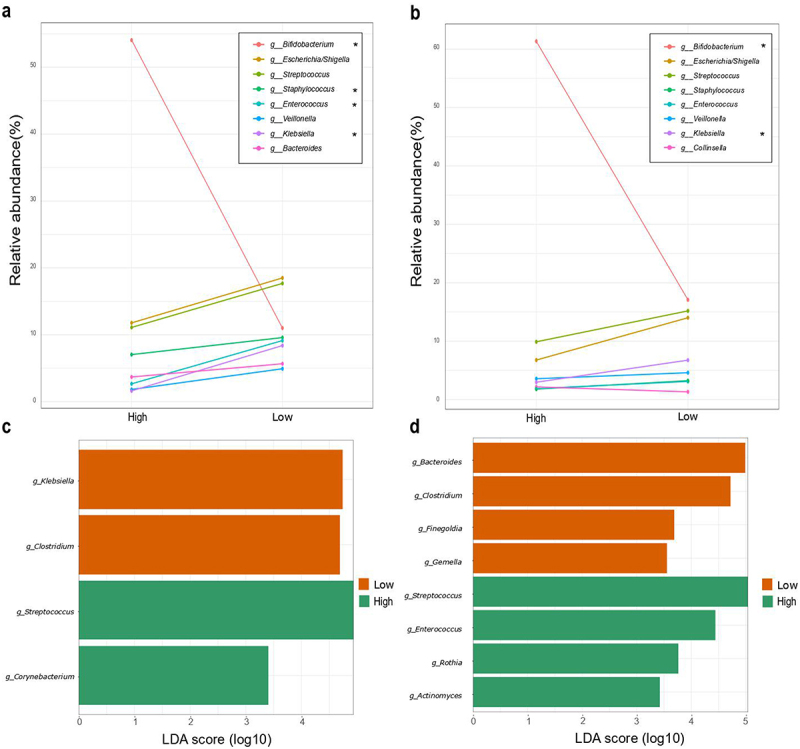
Microbial composition according to *Bifidobacterium*-abundance groups. Differences in relative abundance (%) of the 8 most abundant genera in fecal microbiota (more than 5% of relative abundance) among the first 7 days (a) and 1 month of life (b). Asterisks (*) indicate a significant difference between groups based on Mann–Withney U of the log-transformed relative abundances. Supplementary tables 5 and 6 provide genus abundances and statistical analyses of the groups for the two time points. Linear discriminant analysis (LDA) Effect Size (LEfSE) plot of taxonomic biomarkers identified in the gut microbiota of infants of 7 days (c) and 1 month (d) of age, according to *Bifidobacterium* dominance cluster (High and Low colored in green and orange, respectively). The threshold for the logarithmic discriminant analysis (LDA) score was 3 and *p* < .05 for Wilcoxon test was considered significant.

Differences in relative abundance of the 8 most abundant genera (more than 5% of relative abundance) were calculated according to zOTUs counts (Figure 2(a,b) and Supplementary tables S5 and S6). *Bifidobacterium* relative abundance was 54.38% and 61.72% in the High-clustered samples of 7 days and 1 month respectively, and 11.26% and 17.08% of the Low-clustered samples. *Bifidobacterium* genus was quantified in 7 days old infant samples at 6.93 (3.71) log copies/µL (median (IQR)) for Low cluster and 7.71 (7.03) log copies/µL (median (IQR)) for High cluster. For 1-month samples, 7.08 (4.53) and 7.53 (6.14) log copies/µL (median (IQR)) were quantified in Low and High groups, respectively.

In 7 days old infants, *Streptococcus*, and *Escherichia/Shigella* spp. predominated in the low-*Bifidobacterium* group, appearing in higher relative abundances than the *Bifidobacterium* species, although no statistical differences were found between the two groups. Seven-day-old infants in the low-*Bifidobacterium* group had a significantly higher relative abundance of *Staphylococcus* (*p <* .01), *Enterococcus* (*p <* .05), and *Klebsiella* (*p <* .05), compared to the high-*Bifidobacterium* group. *Veillonella* and *Bacteroides* showed the same pattern but without statistical differences. On the other hand, low-*Bifidobacterium* 1-month-old infants had also higher relative abundance of *Klebsiella* (*p <* .05), whereas *Collinsella* appeared in higher proportions in the high-*Bifidobacterium* group, though differences between groups were not statistically significant. Similar to 7-day-old infants, *Staphylococcus, Enterococcus*, and *Veillonella* appeared in higher abundances in the low-*Bifidobacterium* group, but with no statistically significant difference compared to the high-*Bifidobacterium* group. Tendency analysis of the microbiota also showed that *Bacteroides* spp. was more abundant in high-*Bifidobacterium* infant than in low*-Bifidobacterium* infants at 1 month of age (*p* < .001) (Supplementary table S5 and S6).

LEfSe, performed on samples from 7-day-old infants, showed that *Klebsiella* (*p =* .031) and *Clostridium* (*p =* .0036) were significant characterized in the Low cluster and *Streptococcus* (*p =* .024), and *Corynebacterium* (*p =* .0378) characterized the High cluster. For the 1-month-old infant samples, *Streptococcus* (*p =* .011), *Enterococcus* (*p =* .042), *Rothia* (*p* = .038) and *Actinomyces* (*p =* .021) were enriched in the High cluster, and *Bacteroides* (*p =* .0009), *Finegoldia* (*p* = .009) and *Gemella* (*p =* .045) were enriched in the Low cluster (Figure 2(c,d)).

**Figure 3.**
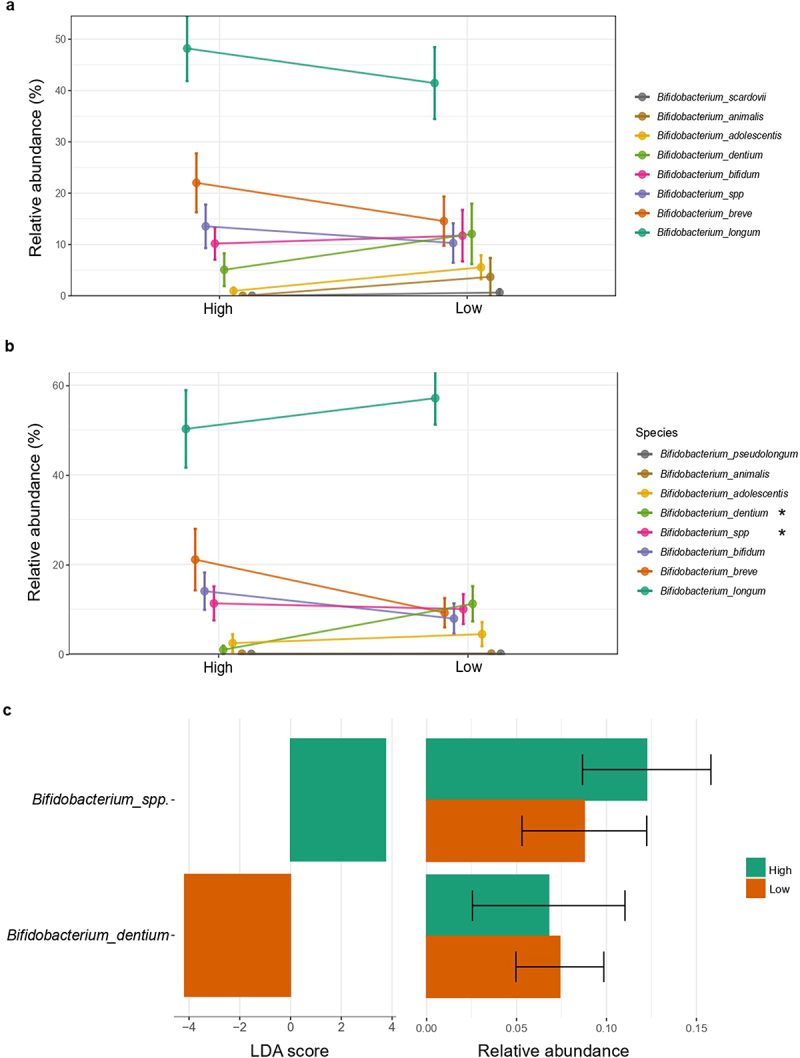
*Bifidobacterium* spp. relative abundance according to *Bifidobacterium relative* abundance group. Differences in relative abundance (%) of species of *Bifidobacterium* identified in fecal microbiota among the (a) first 7 days and (b) 1 month of life. (c) Linear discriminant analysis (LDA) Effect Size (LEfSE) plot of *Bifidobacterium* species identified in the gut microbiome of 1-month-old infants and their relative abundances. Species bacterial traits found at *Bifidobacterium* species level according to *Bifidobacterium* dominance cluster (High and Low coloured in green and orange, respectively). The threshold for the logarithmic discriminant analysis (LDA) score was 3 and *p* < .05 for Wilcoxon test was considered significant.

The Bayesian LCA-based Taxonomic Classification Method for *Bifidobacterium* spp. showed that, in both time points, *Bifidobacterium longum* (High: 46.96–50.72%; Low: 46.09–56.24%, for 7 days and 1 month, respectively) was the most abundant species of *Bifidobacterium* in infants (Figure 3a,b). Relative abundance of *B. dentium* (High: 3.95–10.66%; Low: 13.35–14.48%) was significantly more abundant in 1-month-old infants in the low-*Bifidobacterium* group according to LEfSE analysis (Figure 3c), whereas an unclassified species of *Bifidobacterium* was enriched in the high-*Bifidobacterium* group. This species, together with *B. breve*, appeared in higher amounts in the high-*Bifidobacterium* cluster of infants in both time points, but without statistical significance. On the other hand, *B. adolescentis, B. animalis* and *B. scardovii* were more abundant in low-*Bifidobacterium* infants of both ages, but no significant differences were found. The phylogenetic tree of *Bifidobacterium* species confirmed that, in both time points, there were unique zOTUs assigned for specific *Bifidobacterium* spp. (Supplementary figure S3).

### Influence of environmental factors on Bifidobacterium relative abundance and specific microbial associations

**Figure 4.**
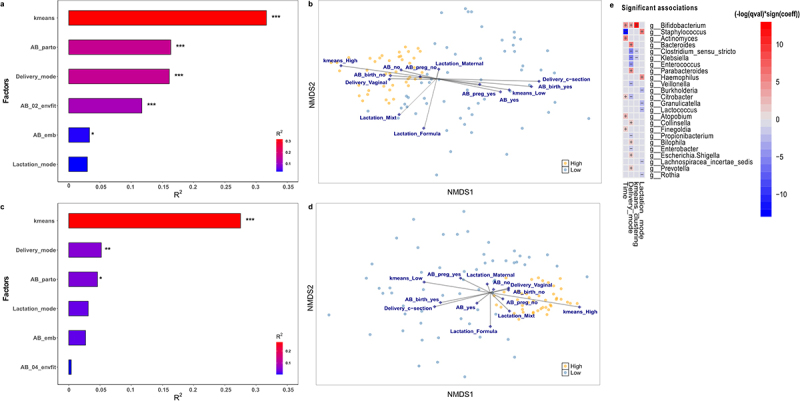
Environmental factors contributing to *Bifidobacterium*-specific microbiota. Significance and explained variance of 6 microbiota covariates modeled by ‘envfit’ across all data types for each time point: for (a) 7 days old and for (c) 1 month old. Horizontal bars show the amount of variance (R^2^) explained by each covariate in the model as determined by ‘envfit’. The groups within each covariate are detailed in Table 1. Covariates are gradient-colored based on R^2^. Significant covariates (false discovery rate (FDR) *p* < .05) are represented in bold. Asterisks (*) denotes the significant covariates at each time point: * *p* < .05, ** *p* < .01, *** *p* < .001. kmeans: hierarchical clusterization based on k-means according to *Bifidobacterium* relative abundance; AB_birth: antibiotic exposure at birth; AB_pregnancy: antibiotic exposure of the mother during pregnancy; AB_exposure: antibiotic exposure of the infant during his life; Delivery_mode: mode of delivery; Lactation_mode: mode of lactation. Dissimilarities in gut microbiota composition according to *Bifidobacterium* relative abundance (High or Low) represented by nonmetric multidimensional scaling (NMDS) of zOTU relative abundances for infants (b) 7 days and (d) 1 month of age. The centroids of each group are in blue (Low) and yellow (High). The top contributing categorical variables are displayed as arrows, with a length proportional to the correlation between the variable and the NMDS ordination. (e) Multivariable association between factors and taxonomy in both clusters while accounting for potentially confounding covariates using MaAsLin.

Analysis with ‘envfit’ confirmed that the high-low *Bifidobacterium* relative *abundance* clustering, based on k-means hierarchical clustering method, is the best approach to explain the dissimilarities in gut microbial composition according to *Bifidobacterium* relative abundance, as it was the factor that explained the largest amount of variance in both time points (R^2^ = 0.316, *p* < .001 at 7 days; R^2^ = 0.275, *p* < .001 at 1 month) (Figure 4(a,c)). In 7-day-old infants, antibiotic exposure at the moment of birth (R^2^ = 0.163, *p* < .001) was the second most important categorical factor, followed by mode of delivery (R^2^ = 0.161, *p* < .001). C-section procedures imply the use of antibiotics during delivery, so it is expected than both variables have similar contributions. Accordingly, vaginal-delivery and absence of antibiotic exposure at birth were associated with the high-*Bifidobacterium* group (Figure 4(b)). Overall antibiotic exposure during the first 7 days of life (including moment of birth till 7 days of age) was the next most important factor (R^2^ = 0.117, *p* < .001), followed by mode of lactation, which was not statistically significant (R^2^ = 0.045, *p* > .05), and the use of antibiotics by the mother during pregnancy (R^2^ = 0.033, *p* < .05).

Delivery mode was the second most important environmental factor contributing to the different microbial composition at 1 month of life (R^2^ = 0.0521, *p* < .01) (Figure 4(c)). Vaginal birth was associated with high-*Bifidobacterium* infants (Figure 4(d)). Lactation mode played a crucial role in the gut composition (R^2^ = 0.051, *p* < .05), even more than antibiotic exposure at birth (R^2^ = 0.046, *p* < .05). Breastfeeding (both, exclusive breastfeeding and mixed practises) contributed to the high-*Bifidobacterium* cluster, while infant formula-feeding was associated with the low-*Bifidobacterium* group. Antibiotic exposure of the mother during pregnancy and of the infant during the first month of life were the least contributing factors, without statistical significance (R^2^ = 0.033, *p* > .05 and R^2^ = 0.001, *p* < .05, respectively).

Significant associations between microbial genera and environmental variables were assessed (Figure 4(e)). The *Bifidobacterium* genus was most strongly and positively correlated with k-means clustering, followed by time of sampling and delivery mode. Hence, these results support the previous analysis, confirming that the microbial composition of our population differentiates into two groups due mainly to *Bifidobacterium* relative abundance. Moreover, *Clostridium* and *Klebsiella* species correlate negatively to this clustering. The variable time shows a significant positive correlation with *Actinomyces, Citrobacter, Atopobium* and *Finegoldia*, while it is negatively correlated with *Staphylococcus*. Mode of delivery is positively associated with *Bacteroides, Parabacteroides, Collinsella, Bilophila, Escherichia/Shigella* and *Prevotella*, and mode of lactation, is positively correlated with *Staphylococcus* and *Haemophilus* genus.

### *Bifidobacterium* relative abundance modulates antibiotic resistant gene load

**Figure 5.**
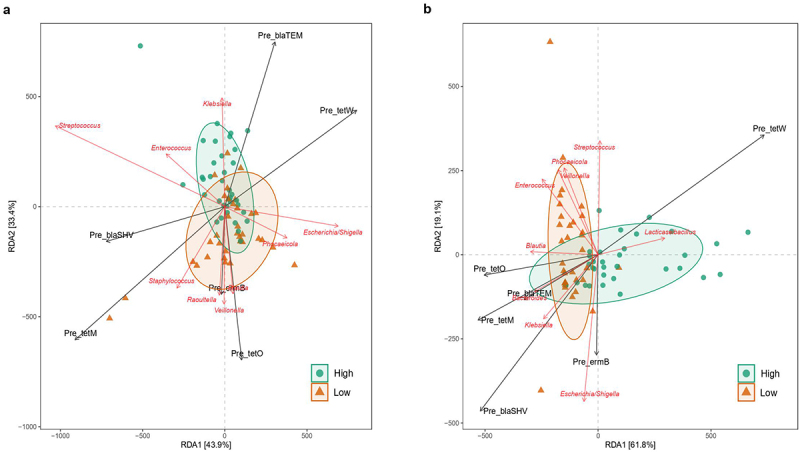
Redundancy Discriminant Analysis (RDA) biplot depicting the relationship between the bacterial communities and copies of ARGs. Coloured arrows represent differential bacteria and copies ARGs, colored dots represent samples from different groups. Angles between the arrows represent correlations; acute angles represent positive correlations and obtuse angles represent negative correlations. The black arrows are the ARGs that best explain the differences between the sample groups. The orange arrows are the taxa that best explain the differences between the sample groups (*n* = 9 taxa).

Antibiotic resistance load and presence diverged depending on *Bifidobacterium* relative abundance (Supplementary table S7). The multivariate redundant discriminant analysis (RDA) (Figure 5) showed that, for 7 days infant gut samples, *tetO, ermB*, and tetM were the ARGs associated with the Low-*Bifidobacterium* cluster, and further analysis suggest that their sources are likely *Escherichia/Shigella, Phocaeicola, Blautia, Veillonella, Raoutella*, and *Staphylococcus*, whereas *Streptococcus, Enterococcus*, and *Klebsiella* were associated to the high-*Bifidobacterium* cluster.

In 1-month-old infants, the Low cluster was positively associated with *Blautia, Enterococcus, Veillonella*, *Phocaelcola, Streptococcus, Escherichia/Shigella, Klebsiella*, and *Bacteroides*, and the last three genera were highly correlated with copies of *tetO, tetM, blaTEM, blaSHV* (*p =* .016), and *ermB* resistant genes. On the contrary, *tetW* is negatively correlated with the other ARGs and taxa and is associated with infants from the high-*Bifidobacterium* cluster. This gene (*tetW*) is more prevalent (*p =* .02) in 1-month-old infants in the high-*Bifidobacterium* (73.08% of presence) group compared to the low-*Bifidobacterium* (42.50%).

**Figure 6.**
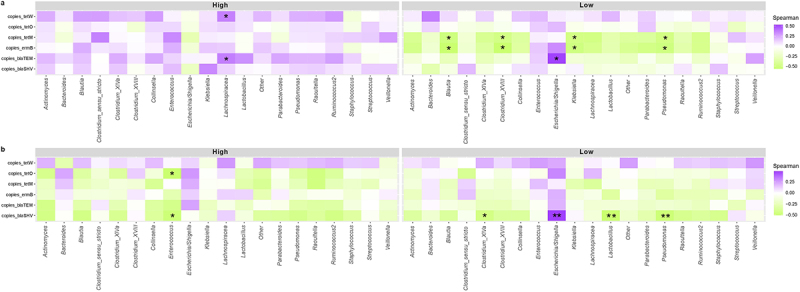
Spearman correlations between genera and copies of ARGs. Heat map to show Spearman correlations between copies of ARGs and microbiota composition at the genus level, divided by *Bifidobacterium* relative abundance clusters (High and Low) for infants of 7 days of age (a) and 1 month of age (b). Statistical differences are marked as follows: * *p* < .05, ** *p* < .01.

Spearman correlations between microbiota at the genus level and copies of ARGs were performed (Figure 6). In 7-day-old infants with higher abundance of *Bifidobacterium*, a high abundance of the *Lachnospira* genus was associated with a higher abundance of *blaTEM*, and *tetW* gene (*p <* .05), while in the low-*Bifidobacterium* infants, a higher abundance of *blaTEM* gene was associated to higher abundance of *Escherichia/Shigella* (*p <* .05). In addition, in the low-*Bifidobacterium* cluster, *tetM*, and *ermB* genes appear negatively correlated with *Blautia, Clostridium, Klebsiella*, and *Pseudomonas* (*p <* .01).

On the other hand, in the High cluster of 1-month-old infants, there is a positive correlation between *blaSHV*,and *tetO* genes and *Enterococcus* (*p <* .01). For the Low cluster, *blaSHV* is negatively correlated with *Clostridium* (*p <* .05), *Lactobacillus* (*p <* .001), and *Pseudomonas* (*p <* .001), and positively correlated with *Escherichia/Shigella* (*p <* .001).

## Discussion

We demonstrated the association of *Bifidobacterium* spp. with the modulation of early-life microbiota and the antibiotic resistance gene load, and determined which perinatal factors are involved in these complex interactions.

Our primary findings showed that the presence of ARGs in infant’s gut correlated with a low-*Bifidobacterium abundance*, so we focused on how the abundance of *Bifidobacterium* could shape the infant resistome in the early stages of life. Results from this resistance epidemiology study align with previous studies^6:^ the microbiota of low-*Bifidobacterium* dominated infants is associated with a higher abundance of antibiotic resistant genes, due to a differential microbial composition responsible for carrying these ARGs, as well as the external factors to which the infant is exposed, such as mode of delivery, antibiotic exposure, and breastfeeding. Overall, our results provide insight into the very early acquisition and development of ARGs in a general population cohort of healthy infants.

### Bifidobacterium relative abundance is associated with a specific gut microbiota

The infant gut resistome changes primarily in relation to the microbiome as ARGs are carried by the microorganisms in the gut and transferred between them through horizontal gene transfer. As far as we know, this is the first study encompassing the influence of *Bifidobacterium* on antibiotic resistance load in infants of 7 days and 1 month of age. These two time points are of high interest as the gut microbiota at these ages is very diverse and still in the early stages of development. Thus, it is still largely shaped by the initial colonizers that the baby is exposed to during delivery and by feeding mode. This is the reason why the specific composition of the gut microbiota can vary widely between individuals.

To achieve our objective, we first studied the microbial profile of our study population. The application of k-means clustering algorithms elucidated that the infants in the study clustered into two different groups according to *Bifidobacterium* spp. abundance. *Bacteroidetes* appeared more abundant in those samples with low abundance of *Bifidobacterium*, enhancing a potential interaction between *Bacteroides* spp. and *Bifidobacterium* spp. Both are generalist glycan degraders capable of using a wide range of substrates: *Bacteroides* spp. are specialized as primary degraders in the metabolism of complex carbohydrates, whereas *Bifidobacterium* spp. more commonly metabolize smaller glycans, such as oligosaccharides, sometimes through syntrophic interactions with *Bacteroides* spp., in which they act as secondary degraders.^38,39^ Some bifidobacteria can produce exopolysaccharides (EPS), which can be metabolized by *Bacteroides*.^40^ It has been described that cocultures of *Bifidobacterium* and *Bacteroides* behave differently against fermentable carbohydrates as a function of the specific characteristics of the strains from each species.^41^

It is also known that *Bifidobacterium* strains have antibacterial activity against various pathogens, including *Escherichia coli, Streptococcus* spp., *Salmonella* spp., *Listeria* spp. and *Cutibacterium* spp,^42^ as well as against multidrug-resistant pathogens,^43^ by nutrient competition,^44^ biofilm removal or inhibition,^45,46^ production of antimicrobial substances,^47,48^ acidification, and enhancement of mucus barrier function.^49^ In addition, *Bifidobacterium* can influence the overall composition and diversity of the gut microbiota, which helps to maintain a diverse and stable microbial community in the gut.^50^ In our study we have observed that *Escherichia/Shigella* and *Streptococcus* are more abundant than *Bifidobacterium* in the low-*Bifidobacterium* cluster of infants at 7 days of life, which reverts at 1 month of life. This may be explained by the impact of breastfeeding on the infant microbiome, as it is well-known that breastfeeding enriches the *Bifidobacterium* dominance,^51,52^ and most of our study population is breastfed. Moreover, at both time points, low-*Bifidobacterium* infants had higher abundances of *Staphylococcus, Klebsiella*, and *Clostridium* than high-*Bifidobacterium* infants. These genera have been previously found to carry ARGs,^2^ therefore, suggesting that the correlation between *Bifidobacterium* and antibiotic resistance load can be a result of the genera that predominate when *Bifidobacterium* are less dominant. All these results highlight the importance of considering specific species and strain interactions in the relationship among intestinal microbial populations and its dynamics over time.

The application of Bayesian LCA-based method in our microbial population showed a distinct *Bifidobacterium* species profile in the two groups. Specifically, 1-month-old infants with low-*Bifidobacterium* abundance were enriched with *Bifidobacterium dentium*, which is found primarily in the oral cavity and possesses various enzymes that enable it to break down complex carbohydrates and degrade protective glycans on mucin proteins.^53,54^ Moreover, *B. dentium* is capable to assimilate type 1 and type 2 HMOs.^55,56^ By fermenting these carbohydrates, *B. dentium* produces SCFAs and other metabolites that provide energy to both itself and other gut bacteria.^57^ This metabolic activity may contribute to the overall functioning and health of the gut ecosystem, enhancing the potential beneficial role of this species in the gut of low-*Bifidobacterium* infants. A non-classified *Bifidobacterium* spp. was enriched in the high-*Bifidobacterium* group of infants, highlighting the importance of species-specific identification in microbial studies to understand the role of each species in the early gut establishment and maturation.

### Antibiotic resistance genes in the infant gut

Antibiotic-resistant bacteria carry a set of antibiotic resistance genes, and this set in a population of bacteria is known as the resistome. Most predominant resistance genes encode for proteins that confer resistance to beta-lactams, tetracyclines, macrolides, aminoglycosides, and quinolones.^17^ Beta-lactamases have been prescribed for many decades, and are commonly used during pregnancy and labor, so it is understandable that resistance appears in infant fecal samples.^2^ Although tetracycline antibiotics are no longer used in pregnancy, *tet* genes have been reported to be the most abundant among infant fecal samples and the most representative resistance gene in the infant resistome.^58,59^ Klassert et. al^2^ determined that genes conferring resistance to macrolides (*mefA, ermB*, and *ermC*) showed the highest dissemination in a sample set of mother-infant pairs and also present the highest likelihood of vertical transmission.^60^

Our primary results showed a lower number of *tetM* copies in the guts of those infants clustered in the high-*Bifidobacterium* group, although, as mentioned, these antibiotics are not used during pregnancy. The prevalence of tetracycline resistance genes in various bacteria species, coupled with the common utilization of tetracycline to treat infections, may explain the acquisition of *tet* genes by women. Although this antibiotic is not used during pregnancy and early life due to potential dental staining effects, it is still extensively used in animals and, therefore, the environment and the diet may be sources of *tet* genes without direct exposure to the antibiotic.^59^ Hence, the vertical transfer of antibiotic resistances from the mother to their offspring, together with the high prevalence of this resistance in the environment, may be the principal mechanism of spread of tetracycline resistance in early life.

To complete the study, other ARGs conferring resistance to antibiotics that are used during pregnancy and labor (beta-lactams and macrolides) were measured and the relationship with specific bacterial communities was assessed. We quantified of *blaTEM, blaSHV*, and *ermB* genes in infant fecal samples, and similarly to *tet* genes, we showed that their presence has been associated with a low abundance of *Bifidobacterium.*

### Antibiotic resistance load is affected by the specific microbiota associated with *Bifidobacterium* relative abundance

Some studies have shown that *Bifidobacterium* can help to reduce the risk of antibiotic resistance by competing with pathogenic bacteria for nutrients and space in the gut and by producing antimicrobial compounds that inhibit the growth of other bacteria.^61,62^ A study conducted in 2019 described a change in the gut microbiome and a reduction in ARG abundance in infants supplemented with *Bifidobacterium infantis* EVC001.^63^ In some studies, the negative correlation between *Bifidobacterium* relative abundance and ARG load has been described in infants from 6 weeks of age.^6^ Moreover, it has been found that *Bifidobacterium* themselves are less likely to possess ARGs than other taxa such as *Enterococcus, Streptococcus, Staphylococcus*, and *Bacteroides*.^6,7^ Pseudomonadota, more specifically, Gammaproteobacteria, have been demonstrated to be the major carriers for ARGs in studies either employing culture-based or metagenomics methods.^64^ Members of the family Enterobacteriaceae, such as *Escherichia, Klebsiella, Citrobacter, and Enterobacter*, which are responsible for a large range of infections, contribute the most abundant and unique ARGs in the infant’s gut.^2^

Comparably, our results showed that at 7 days of age, *tetO, ermB*, and *tetM* are positively correlated with the bacterial species predominate in the Low cluster of infants, namely *Escherichia/Shigella, Phocaeicola, Blautia, Veillonella, Raoutella*, and *Staphylococcus. Streptococcus, Enterococcus*, and *Klebsiella* were associated with the high-*Bifidobacterium* cluster at 7 days old gut microbiota and with the low-*Bifidobacterium* cluster at 1-month old. This result shows the gain of stability of gut microbiota at 1 month of age compared to 7 days, as it has been previously described.^65^

Moreover, at 1 month of age, low-*Bifidobacterium* infants are associated with *Blautia, Veilonella*, *Phocaelcola, Escherichia/Shigella, Klebsiella*, and *Bacteroides*, the last three of which highly correlated with copies of *tetO, tetM, blaTEM, blaSHV*, and *ermB* resistant genes.

It is important to recall that *tetW* was negatively correlated with the other ARGs and associated with infants from the high-*Bifidobacterium* cluster at this time point, which matches with previous findings that indicate that *tetW* gene is an ARG from the *Bifidobacterium* genus.^66,67^ Spearman correlations also highlighted the influence of *Escherichia/Shigella* on antibiotic resistance load: in low-*Bifidobacterium* infants, this genus was positively correlated with the *blaTEM* resistance gene.

Overall, these findings suggest that low levels of *Bifidobacterium* may lead specific ARG-carrying genera to dominate the infant’s gut, thus increasing the antibiotic resistance load. But how does this *Bifidobacterium* variation occur? Resistome composition is strongly influenced by the phylogenetic profile of the bacterial population. Considering that there are still numerous bacteria possessing unidentified ARGs, the known ARGs may represent only a small part of the true resistome. In this vein, it would be interesting to include genes conferring resistance to other classes of antibiotics, such as aminoglycosides or quinolones, that, together with a metagenomic approach, would provide a wider view of the infant resistome. Aminoglycosides are widely used for neonatal sepsis and are usually combined with beta-lactams,^68^ and resistance to quinolones also emerged with clinical use and has become common in some bacterial pathogens. Although quinolones are in limited in the pediatric population, the presence of ciprofloxacin-resistant *E. coli* in the infant’s fecal microbiota has been described.^69^

### Factors influencing infant’s gut microbiota

High *Bifidobacterium* dominance in infants’ guts has been directly related to the mode of delivery: vaginal or c-section. C-section-born infants have a decreased abundance of *Bifidobacterium* compared to vaginally born infants.^70,71^ For this reason, we assessed whether our microbial clusters may be a result of the procedure of delivery, but we again obtained two different clusters according to *Bifidobacterium* relative abundance, independent of the mode of delivery. All infants born vaginally had a significantly different microbiota depending on if they had high or low *Bifidobacterium* relative abundance, at both 7 days and 1 month of age, so we could discard that mode of delivery was shaping our microbiota clusters. Indeed, k-means hierarchical clustering was the variable with the highest contribution to the separation of the microbial composition of our population, as expected. However, we observed role of delivery in the modulation of gut microbiota, as it was associated with genera such as *Enterococcus, Klebsiella*, and *Clostridium*, which have been previously related to the microbiota of c-section born infants.^72^

In addition, it has been seen that early-life antibiotic treatment disrupts the proper and natural development of gut microbiota with a potential negative influence on later health.^73^ It has also been previously described that intrapartum antibiotic prophylaxis (IAP) has a negative effect on the later bifidobacterial establishment in the neonatal gut.^7,74^ To assess this effect, antibiotic exposure of our cohort was recorded during pregnancy and also during the first month of life of the infants. During a c-section procedure, antibiotics are commonly administered as a preventive measure to reduce the risk of infection (e.g., cefazolin, vancomycin, clindamycin and erythromycin).^75^ On the other hand, there is the possibility that vaginal-born infants have been exposed to antibiotics at birth too, and this has been recorded and considered in our study. Interestingly, the antibiotic exposure at delivery and how childbirth occurred had a similar contribution to the microbial composition of infants of 7 days of age, while this interaction dissociated at 1 month of age, with the antibiotic exposure losing importance, indicating a possible mitigation of its effects over time. Nevertheless, in both time points, c-section procedures and exposure to antibiotics contributed to a gut microbiota with low *Bifidobacterium* spp. Therefore, our results confirm previous knowledge on the effect of antibiotics on the early microbial composition but highlight the importance of *Bifidobacterium* abundance in the infant’s gut.^76^

The feeding mode showed a significant contribution to 1-month-old infants’ microbiota but not to that at 7 days old, maybe because of the short period infants consumed milk or because a reduced number of infants that where mixed or formula-fed. Formula-feeding contributed to the low-*Bifidobacterium* microbiota profile, while mixed and exclusive breastfeeding contributed to the high-*Bifidobacterium* infants, highlighting the widely known benefits of breastfeeding practises for infant health ,^77 ,^ .^78,79^ Nevertheless, breastmilk plays a crucial role on the acquisition of antibiotic resistance in early life.^80^ Human milk has been found to contain antibiotic-resistant bacteria, such as *Staphylococcus, Streptococcus, Acinetobacter, Enterococcus*, and *Corynebacterium*, resistant to antibiotics and with multidrug-resistant profiles.^81^ Moreover, metagenomic analyses have highlighted the high levels of ARGs and MGEs in breastmilk and the similarity of the breastmilk and infant gut resistomes.^3^ Overall, more understanding on mother’s gut and breastmilk resistomes may shed light to decipher how neonates acquire antibiotic resistance genes in early life and the role that environmental factors may play, so new approaches and decisions can be made to reduce antibiotic resistance. The interplay between early microbial colonization, environmental factors, and dietary changes might have lasting effects that extend beyond infancy. Understanding the persistence of these early exposures and their potential link to ARGs could offer valuable insights into strategies for promoting healthy gut microbiota development and combating antibiotic resistance.

Our results support the notion that microbiota plays a crucial role in early-life antibiotic resistance gene acquisition, focusing on infants of 7 days and 1 month of age. *Bifidobacterium* dominance has been found to be associated with the load of ARGs: low-*Bifidobacterium* abundance is associated with a higher presence of ARGs in infant’s gut, which is also associated with a specific microbial profile. Moreover, we assessed that antibiotic exposure and mode of delivery are two of the key factors influencing microbial composition, and thus, antibiotic resistance load, and we suggest the potential impact of new or current antibiotic prescription practices during pregnancy, at birth, or during the first month of life on the infant gut microbiome and resistome. Our findings highlight the public health concern of antibiotic resistance and elucidate that more studies are needed to gain further insights into how prevalent antibiotic resistance can be in the population and to develop new strategies to ameliorate the ARG load.

## Supplementary Material

Supplemental Material

## Data Availability

The dataset supporting the conclusions of this article is included in the NCBI’s Sequence Read Archive (SRA) repository in the MAMI BioProject ID PRJNA614975 (http://www.ncbi.nlm.nih.gov/bioproject/61497).

## Abbreviations

ARG: Antibiotic Resistant Genes
HMO: Human Milk Oligosaccharides
