# Supplementary figures and images for: The role of Bifidobacterium genus in modulating the neonate microbiota: implications for antibiotic resistance acquisition in early life
Source: Gut Microbes. 2024 May 26;16(1):2357176. doi: 10.1080/19490976.2024.2357176 (PMC11135851; doi:10.1080/19490976.2024.2357176)

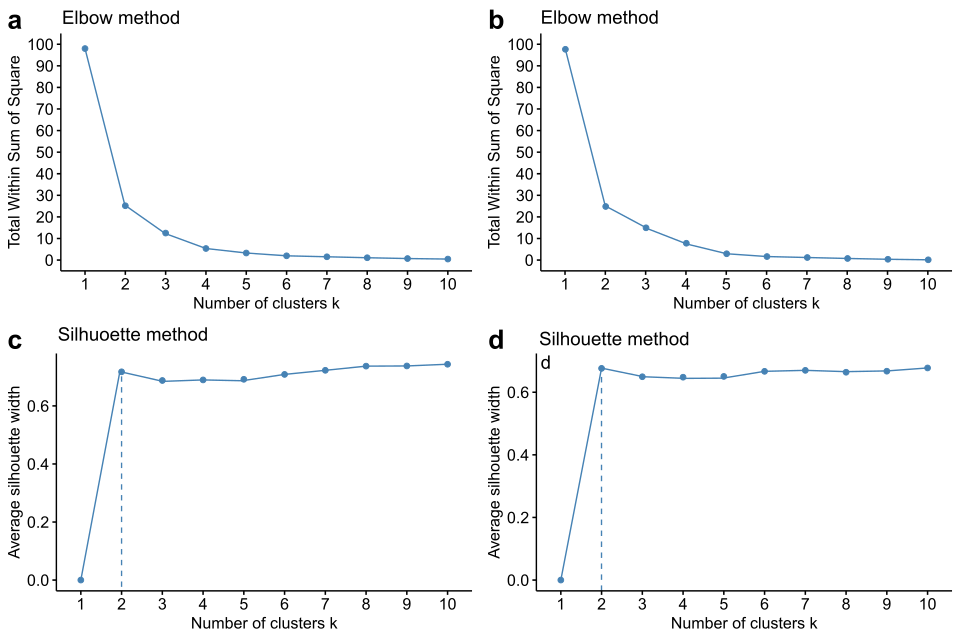

Supplement: Supplemental Material [file KGMI_A_2357176_SM6035.zip › supl_2_elbow.png]

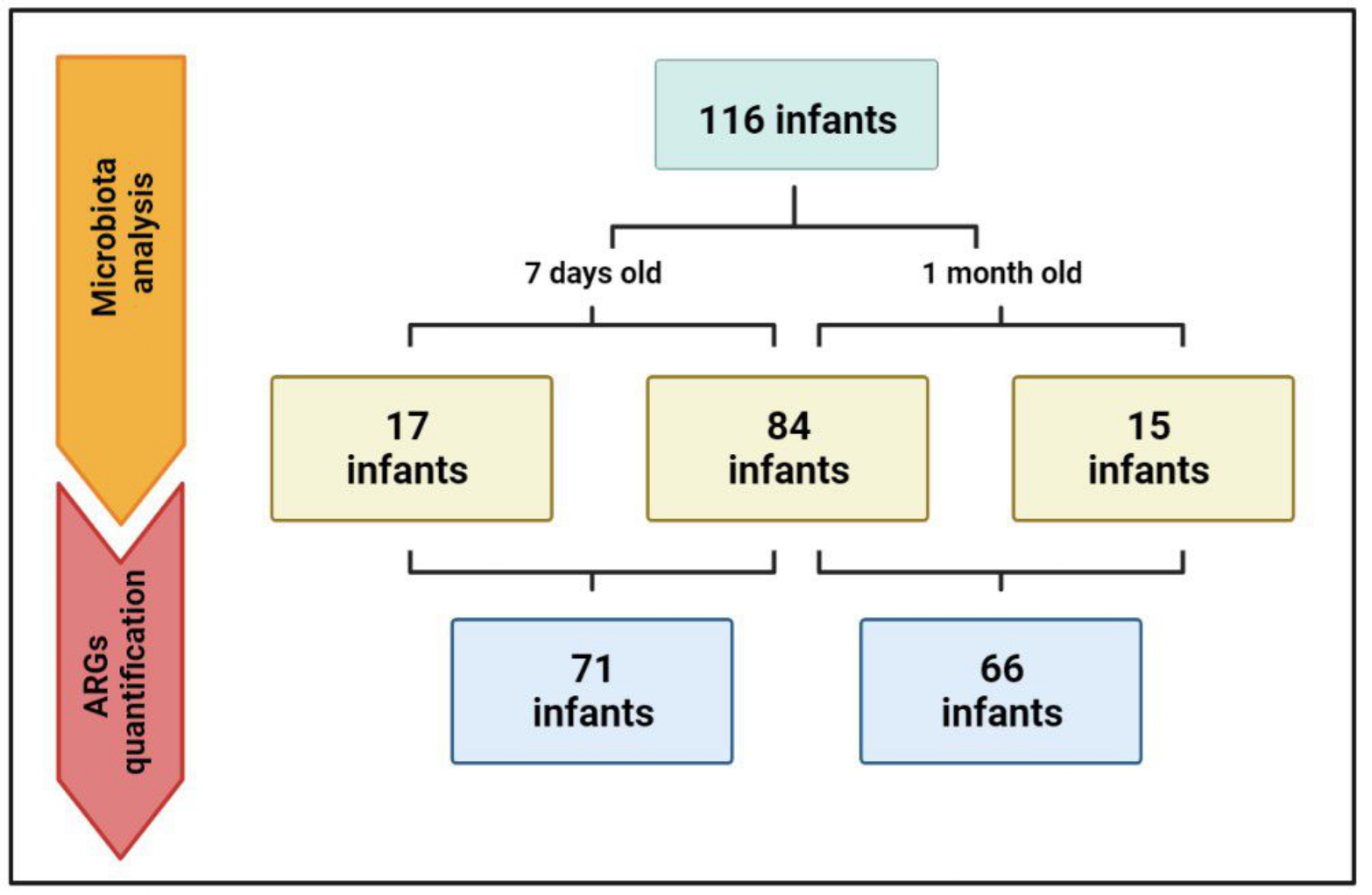

Supplement: Supplemental Material [file KGMI_A_2357176_SM6035.zip › supl_figure_1_flowchart.jpg]

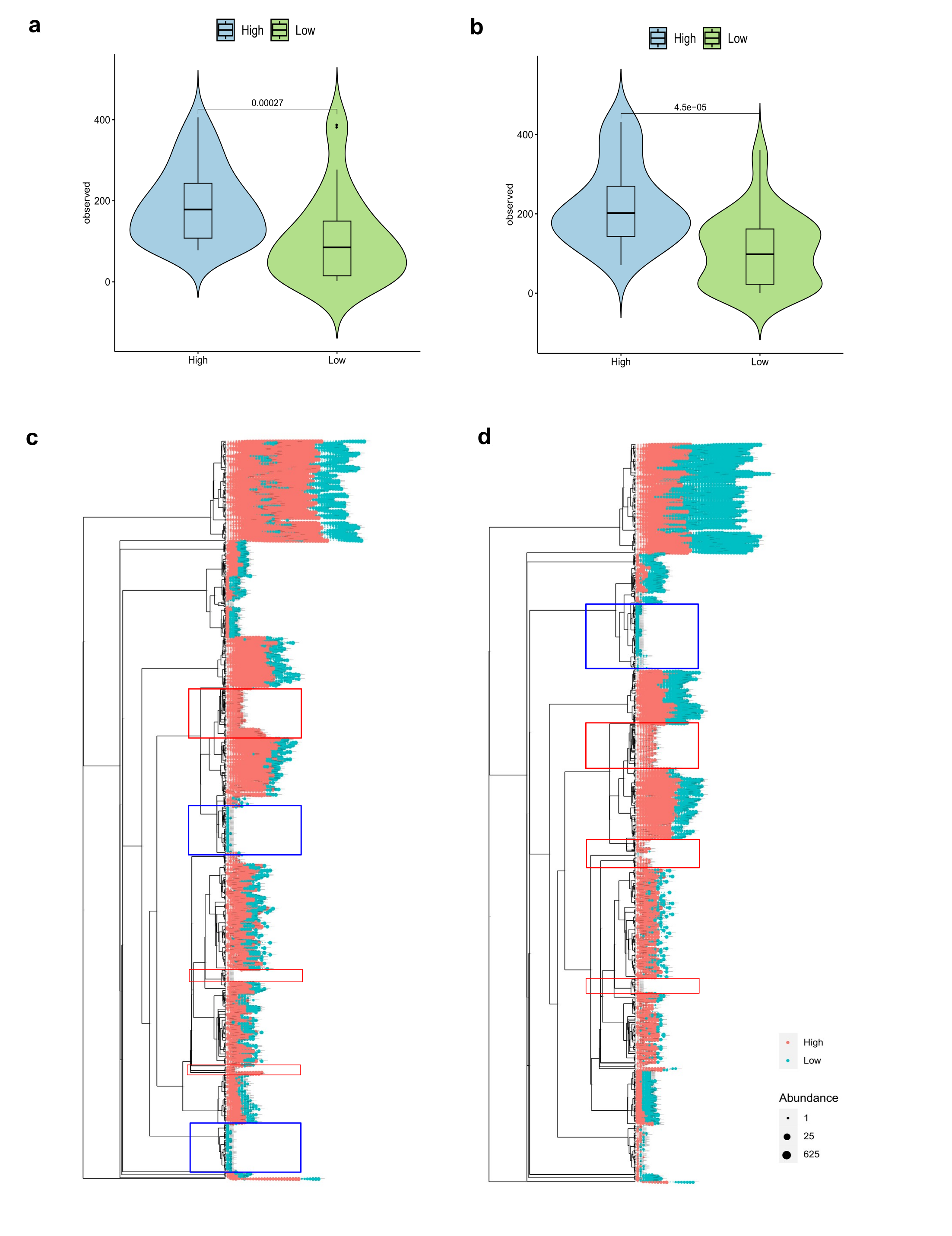

Supplement: Supplemental Material [file KGMI_A_2357176_SM6035.zip › supl_figure_3.png]
